# Supplementary figures and images for: Proteomic characteristics reveal the signatures and the risks of T1 colorectal cancer metastasis to lymph nodes
Source: eLife. 2023 May 9;12:e82959. doi: 10.7554/eLife.82959 (PMC10234629; doi:10.7554/eLife.82959)

## Slide 1
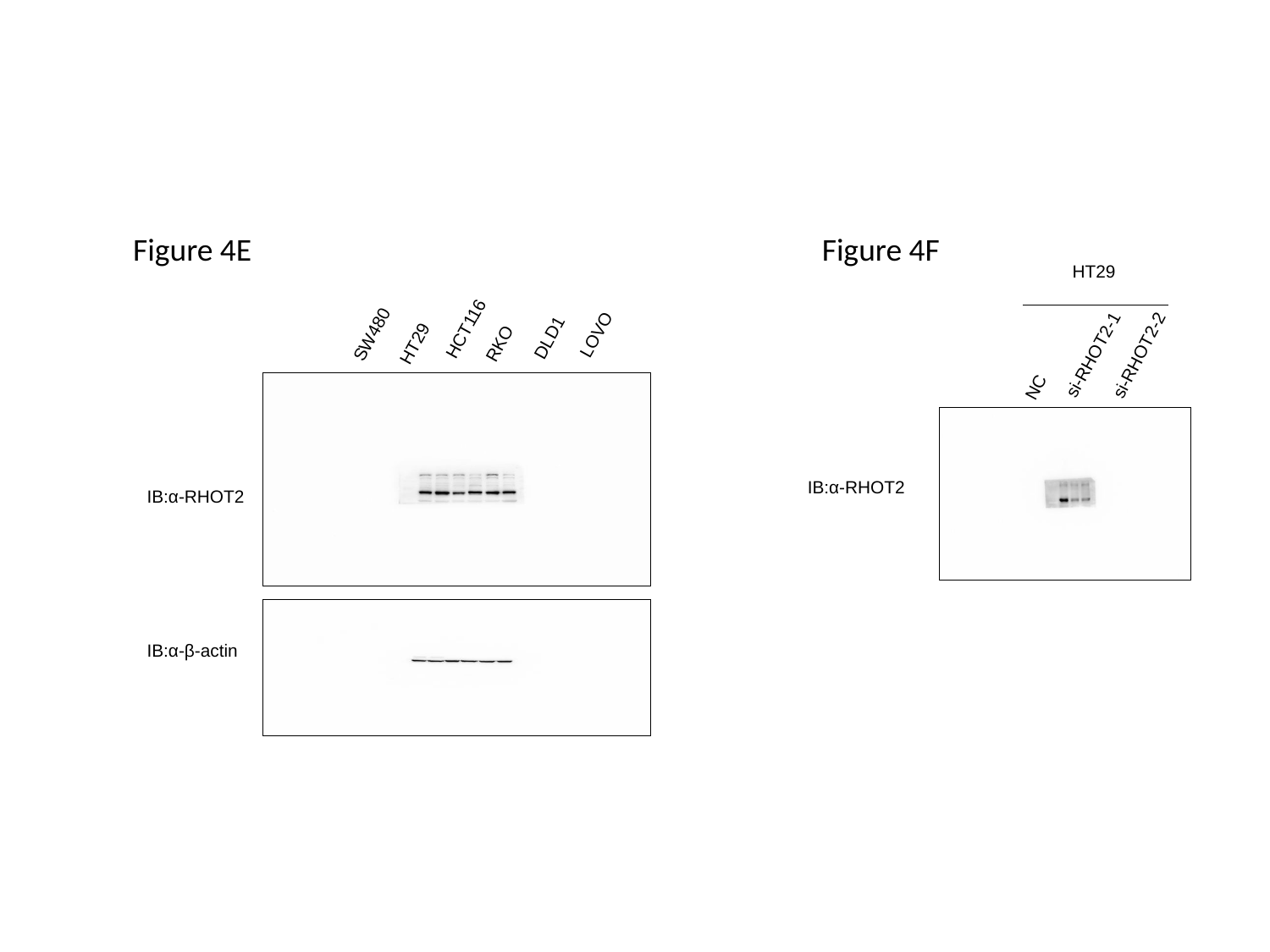

Figure 4E
Figure 4F
HT29
si-RHOT2-1
si-RHOT2-2
NC
IB:α-RHOT2
HCT116
LOVO
SW480
DLD1
HT29
RKO
IB:α-RHOT2
IB:α-β-actin

Supplement: Figure 4—source data 2. [file elife-82959-fig4-data2.zip › Figure4-source data2/Figure4-WB source images.pptx]

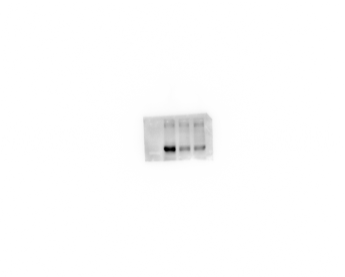

Supplement: Figure 4—source data 2. [file elife-82959-fig4-data2.zip › Figure4-source data2/WB_Figure4F.png]

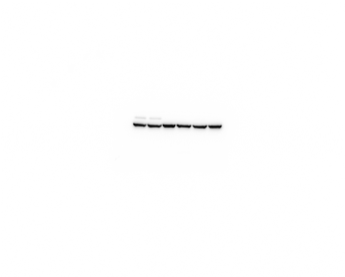

Supplement: Figure 4—source data 2. [file elife-82959-fig4-data2.zip › Figure4-source data2/WB_Figure4E_actin.png]

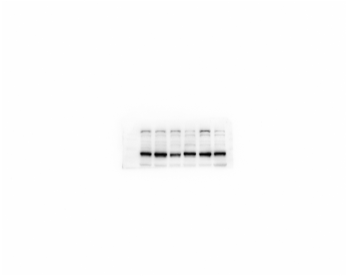

Supplement: Figure 4—source data 2. [file elife-82959-fig4-data2.zip › Figure4-source data2/WB_Figure4_RHOT2.png]
